# Supplementary material for: Helium in diamonds unravels over a billion years of craton metasomatism
Source: Nat Commun. 2021 May 11;12:2667. doi: 10.1038/s41467-021-22860-3 (PMC8113505; doi:10.1038/s41467-021-22860-3)
Supplement: Supplementary file 1 — Supplementary Information [file 41467_2021_22860_MOESM1_ESM.pdf]

Supplementary Information:

Helium in diamonds unravels over a billion years of craton metasomatism

Yaakov Weiss[1,2*], Yael Kiro[3,2], Cornelia Class[2], Gisela Winckler[2,4], Jeff W. Harris[5], Steven L. Goldstein[2,4]

[1] The Freddy and Nadine Herrmann Institute of Earth Sciences, The Hebrew University of Jerusalem, Jerusalem 91904, Israel

[2] Lamont-Doherty Earth Observatory of Columbia University, Palisades, New York 10964, USA

[3] Department of Earth and Planetary Sciences, Weizmann Institute of Science, Rehovot 76100, Israel

[4] Department of Earth and Environmental Sciences, Columbia University, Palisades, New York 10964, USA

[5] School of Geographical and Earth Sciences, University of Glasgow, Glasgow, G12 8QQ, UK

corresponding author: [*]yakov.weiss@mail.huji.ac.il

High-density supercritical fluid (HDF)-bearing diamonds, like the majority of different diamond types, form in the continental lithospheric mantle (CLM) during metasomatic enrichment events by carbon- and water-rich (C-O-H) fluids[1,2]. Constraining the timing and nature of these events is an ongoing challenge in our understanding of the CLM history, and depends on our ability to constrain the age of HDF-bearing diamonds. To date, the limited available Sr-Nd-Pb isotopic data on HDF-bearing diamonds do not offer straight-forward age significance[3-5]. The incompatible element-enriched nature of the included HDFs, alternatively, provide the opportunity to obtain (U-Th)/He radiogenic ages for their host diamond and constrain the timing of C-O-H metasomatic events in the CLM.

The budget of He in HDF-bearing diamonds

Possible sources contributing to the budget of He in an HDF-bearing diamond are: (1) initial He trapped in the diamond lattice during formation; (2) implantation of ^4He from the surrounding host rock; (3) ^3He produced within the diamond lattice from cosmic ray impact; (4) *in situ* radiogenic production of ^4He from U and Th decay and ^3He from $^6Li(n,\alpha)^3H \rightarrow {}^3He$, and (5) ^4He and ^3He trapped within inclusions. ^4He can be lost from the diamond due to recoil from the outermost <25 μm of a diamond, and both ^4He and ^3He can be lost by diffusion. The budget of He in a diamond can therefore be written as:

$$^4He_{Total} = {}^4He_{Diamond\ lattice\ initial} + {}^4He_{Implanted} + {}^4He_{Radiogenic} + {}^4He_{Inclusions} - {}^4He_{Recoil} - {}^4He_{Diffusion}$$

and –

$$^3He_{Total} = {}^3He_{Diamond\ lattice\ initial} + {}^3He_{Cosmogenic} + {}^3He_{Nucleogenic} + {}^3He_{Inclusions} - {}^3He_{Diffusion}$$

The following paragraphs discuss the possible processes that can add He to or remove He from diamonds, and their significance for determining the He content, isotopic compositions and (U-Th)/He age determinations of the De Beers Pool and Finsch HDF-bearing diamonds that were analyzed in the present study.

Contributions of He from the diamond lattice: Release of He (both ^3He and ^4He) from the diamond lattice is possible upon diamond graphitization at ~2000 °C. Kurz et al.[6] showed that the amount of ^4He released from gem-quality monocrystalline diamonds by crushing is ~2 orders of magnitude lower than by burning the same diamond. To evaluate the contribution of He released from the diamond lattice by our sequential crushing method, we crushed and analyzed gem-quality diamonds and found that the amounts of He released are within blank levels, and 2-3 orders of

magnitude lower compared to the De Beers Pool and Finsch HDF-bearing diamonds analyzed. Thus, He trapped in the diamond lattice has a minimal contribution to the budget of He released by crushing HDF-bearing diamond.

Implantation of ^{4}He from the surrounding host rocks: Implanted ^{4}He (α-particles) produced from radioactive decay of U and Th in the surrounding rock can penetrate <25 µm into a diamond[7]. The damage to the diamond lattice is expressed as a unique green and brown surficial coloration[8,9]. The De Beers Pool and Finsch HDF-bearing diamonds have no visual radiation color-change. Moreover, in the present study, inner fragments of the diamond samples were selected for analyses to avoid the possible contributions of implanted ^{4}He from the outermost 25 µm.

Cosmogenic ^{3}He: Spallation reactions that produce ^{3}He in rocks occur predominantly within a few meters of the Earth's surface[10,11]. ^{3}He production by spallation in diamonds is therefore significant only within the upper few meters of the kimberlite diatreme or in alluvial diamonds. On this basis, extremely high ^{3}He/^{4}He ratios (>100 Ra) obtained in alluvial diamonds have been interpreted to result from *in situ* cosmogenic ^{3}He production at the surface[12,13]; a connection that was later confirmed[14]. The studied De Beers Pool and Finsch HDF-bearing diamonds are from deep mining and are therefore unaffected by cosmic ray spallation.

In situ production of ^{4}He and ^{3}He in the diamond lattice: ^{4}He can be produced *in situ* from U and Th decay within the diamond lattice. For a U content of 1 ppb and Th/U ratio of 3.5 in a diamond, only ~10^{-7} ccSTP g^{-1} ^{4}He will be produced over ~4.5 Ga[15], an amount which is 1-2 orders of magnitude lower than the measured concentrations in HDF-bearing diamonds. Moreover, given more realistic U and Th concentrations of 0.15 and 0.45 ppb, respectively, in an inclusion-free diamond[16], the ^{4}He contribution is negligible. ^{3}He can be produced within the diamond by

$^{6}Li(n,\alpha)^{3}H \rightarrow {}^{3}He$. Kurz et al.[6] calculated that over 90 Ma, an amount of $^{3}He=3\times10^{-13}$ cc g^{-1} will be produced within a diamond containing 1 ppm Li, from the production of 2.5×10^{-6} neutrons by Th and U decay within the kimberlite (assuming 5 ppm U and Th/U=3.5 in the kimberlite). Such an amount of nucleogenic ^{3}He is ~2 orders of magnitude lower than most of our measured ^{3}He contents in De Beers Pool and Finsch HDF-bearing diamonds. Moreover, the amount of Li in diamonds rarely exceeds ~200 ppb[17], thus a concentration of 1 ppm Li in a diamond is unrealistically high, and the amount of ^{3}He produced by $^{6}Li(n,\alpha)^{3}H \rightarrow {}^{3}He$ will be much smaller over much longer periods of time.

Loss of ^{4}He from the microinclusions due to α-recoil: ^{4}He produced by U-Th radioactive decay are expected to move <25 µm within a diamond[7]. Thus, radiogenic ^{4}He atoms produced within µm-size C-O-H microinclusions can be implanted into the surrounding diamond matrix. However, the fact that radiogenic $^{3}He/^{4}He$ signatures are observed in HDF-bearing diamond measurements by crushing (Fig. 1; Supplementary Data 1) indicates that these radiogenic ^{4}He atoms find their way back to the microinclusions. A reasonable explanation for such return is fast diffusion of He through radiation-damaged diamond paths[18-20], compared to the very slow diffusion in an ordered diamond lattice (diffusion of He in diamonds is discussed below). While radiogenic ^{4}He will be lost from microinclusions located in the outer <25 µm of the diamond, using inner fragments of the De Beers Pool and Finsch HDF-bearing diamonds for He analyses avoids such a loss.

Diffusive loss of He from diamonds: Luther and Moore[18] were the first to experimentally measure He diffusion in diamonds. Using He-irradiated synthetic diamonds they determined the diffusion coefficient (*D*) to range between 10^{-5}-10^{-7} cm^{2} s^{-1}. These fast diffusion rates result from the radiation-induced damage to the diamond lattice[19,20]. A set of industrial-grade diamonds

yielded slower diffusion rates (D=1.9×10^{-16} cm^2 s^{-1} [19], similar to diffusion rates determined in carbonado diamonds (D=3×10^{-17} cm^2 s^{-1} at 1300 °C[21]) and chemical vapor deposition (CVD) polycrystalline diamonds[22]). Much smaller D-values were determined for He in monocrystalline diamonds at 1000-1300 °C (1-4×10^{-21} cm^2 s^{-1} [23]). Diffusion coefficients in HDF-bearing diamonds have not been determined experimentally, however, considering the large range of possible He diffusivities, between D=10^{-16} to 10^{-21} cm^2 s^{-1}, this process may impact the diamond's potential to retain He over geological time scales and thus can influence its He content, isotopic compositions and resolution of calculated (U-Th)/He ages.

Considering the above, the He budget for the studied De Beers Pool and Finsch HDF-bearing diamonds, which are from deep mining, and whose inner fragments were crushed rather than burned for He analyses, can be therefore simplified to:

$$^{4}He_{Total}={}^{4}He_{Inclusions}-{}^{4}He_{Diffusion}$$

and –

$$^{3}He_{Total}={}^{3}He_{Inclusions}-{}^{3}He_{Diffusion}$$

This He budget is consistent with previous conclusions that the prime source of He in HDF-bearing diamonds is the microinclusions[24-26].

Supplementary Figures

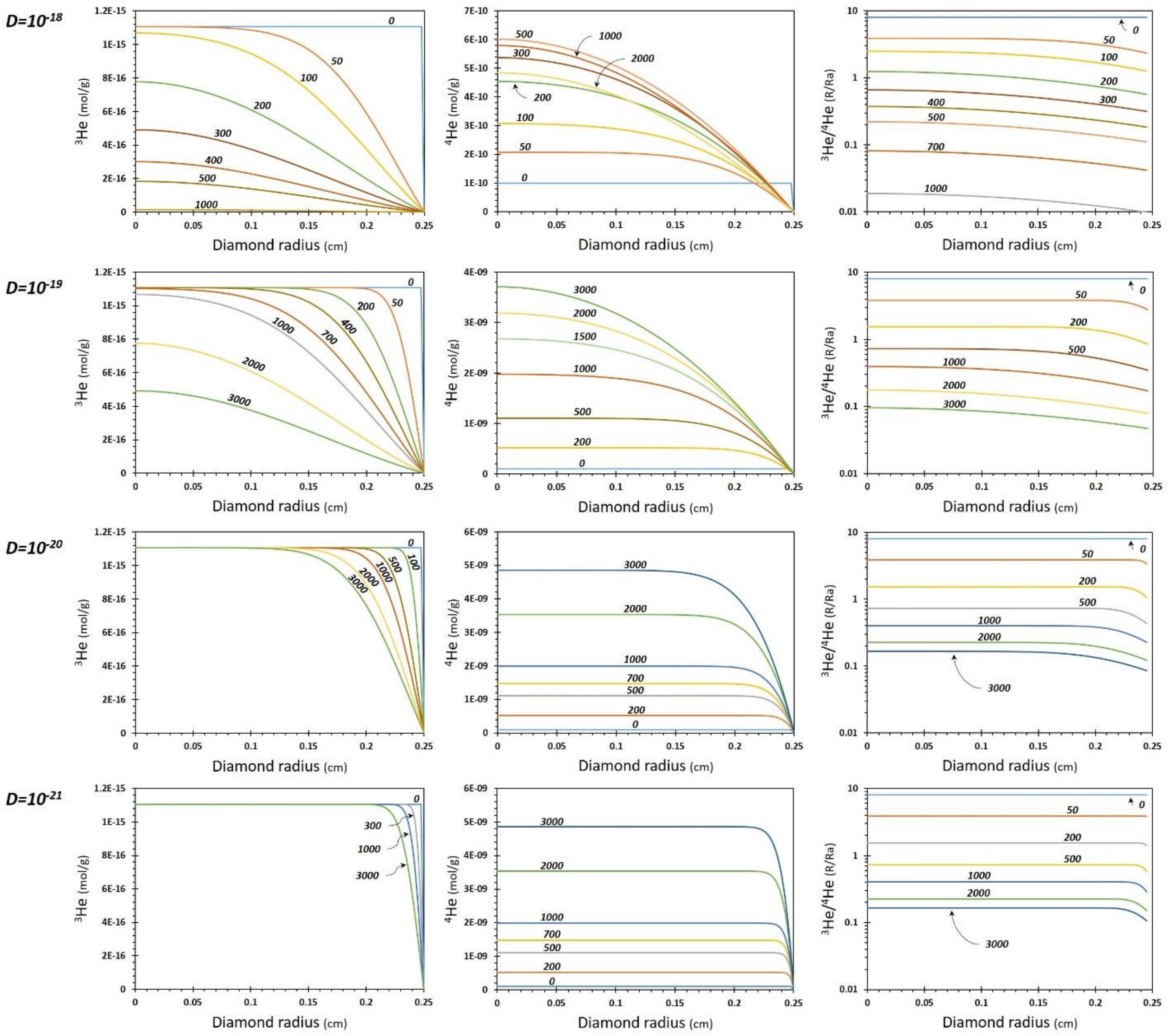

Supplementary Fig. 1: Helium diffusion profiles in a diamond as a function of time for different He diffusivities. The change in ^{4}He and ^{3}He content and ^{3}He/^{4}He isotopic ratio is presented for D=10^{-21}–10^{-18} cm^{2} s^{-1}. Different colored curves are for different ages, in Ma, equivalent to diamond formation age. Model details are in Methods section – 'Diffusion model of He in diamonds'. The parameters used for all calculations: r=2.5 mm, ^{3}He$_{i}$=1.1×10^{-15} mole g^{-1}, ^{4}He$_{i}$=1×10^{-10} mole g^{-1}, U=5×10^{-10} mole g^{-1}, and Th/U=4. These parameters are representative for the studied De Beers Pool and Finsch diamonds and available HDF-bearing diamonds data[26,27].

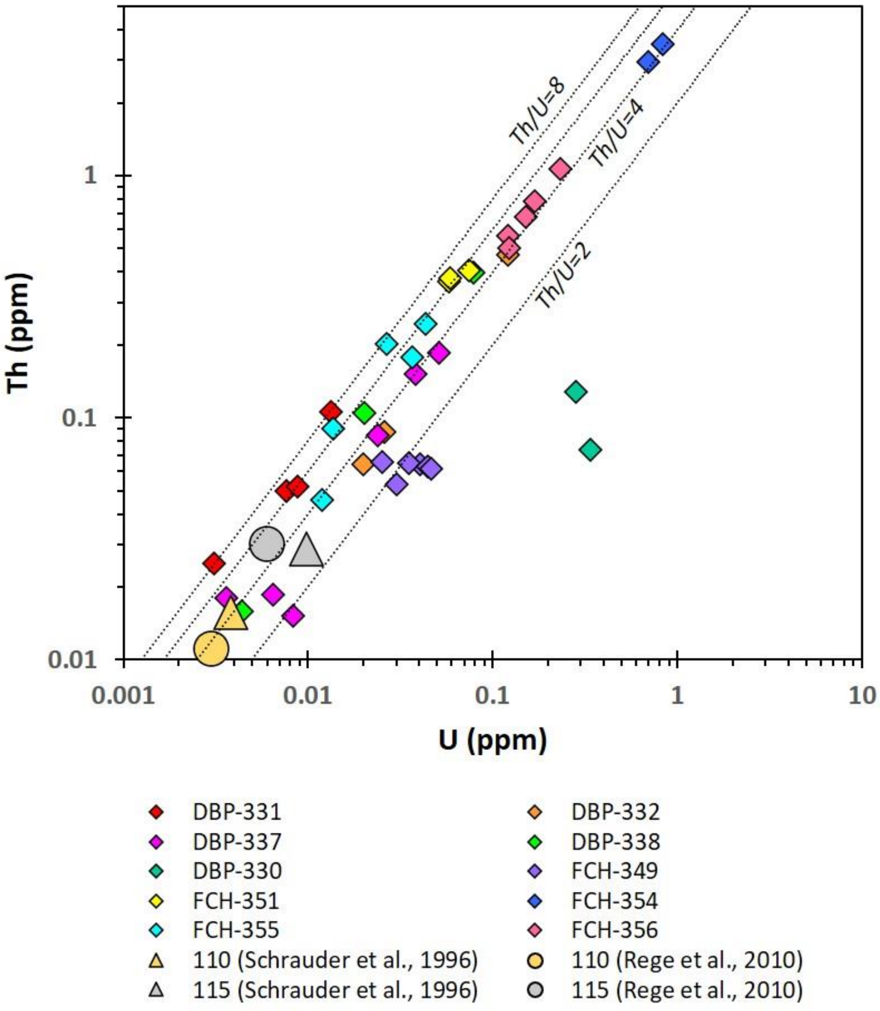

Supplementary Fig. 2: U and Th concentrations in HDF-bearing diamonds from De Beers Pool and Finsch mines. Small diamond symbols coded by color represent duplicate LA-ICP-MS analyses in different microinclusions-bearing parts of the studied diamonds. The variations in U and Th concentrations in a single diamond reflect variations in the spatial abundance of microinclusions, which is small for some of the De Beers Pool and Finsch HDF-bearing diamonds and somewhat larger for others. The conservative Th/U ratios for changing U and Th concentrations demonstrate the compositional homogeneity of the trapped HDFs within a single diamond. Also shown are two diamonds from Jwaneng (samples 110 and 115) that were analyzed multiple times by LA-ICP-MS[28] (average compositions are shown) and by INAA[29], which average a much larger diamond volume (and are considered as bulk analyses). The similarity between the results of the two methods indicates that the bulk trace element composition of HDF-bearing diamond can be closely represented by averaging a few laser ablation ICP-MS analyses of different parts in the diamond.

References

1 Stachel, T. & Luth, R.W., Diamond formation — Where, when and how? *Lithos* **220–223**, 200-220 (2015).

2 Jablon, B. M. & Navon, O. Most diamonds were created equal. *Earth and Planetary Science Letters* **443**, 41-47, doi:http://dx.doi.org/10.1016/j.epsl.2016.03.013 (2016).

3 Klein-BenDavid, O., Pearson, D.G., Nowell, G.M., Ottley, C., McNeill, J.C.R., Logvinova, A., Sobolev, N.V. The sources and time-integrated evolution of diamond-forming fluids – Trace elements and isotopic evidence. *Geochimica et Cosmochimica Acta* **125**, 146-169 (2014).

4 Smith, E.M., Kopylova, M.G., Nowell, G.M., Pearson, D.G., Ryder, J. Archean mantle fluids preserved in fibrous diamonds from Wawa, Superior craton. *Geology* **40**, 1071-1074 (2012).

5 Weiss, Y., McNeill, J., Pearson, D.G., Nowell, G.M., Ottley, C.J. Highly saline fluids from a subducting slab as the source for fluid-rich diamonds. *Nature* **524**, 339-342 (2015).

6 Kurz, M. D., Gurney, J. J., Jenkins, W. J. & Lott, D. E. Helium isotopic variability within single diamonds from the Orapa kimberlite pipe. *Earth and Planetary Science Letters* **86**, 57-68, doi:https://doi.org/10.1016/0012-821X(87)90188-9 (1987).

7 Ziegler, J. F. & Biersack, J. P. in *Treatise on heavy-ion science* 93-129 (Springer, 1985).

8 Hamilton, J. G., Putnam, T. M. & Ehrmann, M. L. Effect of heavy charged particle and fast neutron irradiation on diamonds. *American Mineralogist* **37**, 941-949 (1952).

9 Nasdala, L. *et al*. Radio-colouration of diamond: a spectroscopic study. *Contributions to Mineralogy and Petrology* **165**, 843-861, doi:10.1007/s00410-012-0838-1 (2013).

10 Kurz, M. D. In situ production of terrestrial cosmogenic helium and some applications to geochronology. *Geochimica et Cosmochimica Acta* **50**, 2855-2862, doi:https://doi.org/10.1016/0016-7037(86)90232-2 (1986).

11 Lal, D. Production of 3He in terrestrial rocks. *Chemical Geology: Isotope Geoscience Section* **66**, 89-98 (1987).

12 McConville, P. & Reynolds, J. Cosmogenic helium and volatile-rich fluid in Sierra Leone alluvial diamonds. *Geochimica et Cosmochimica Acta* **53**, 2365-2375 (1989).

13 Lal, D., Craig, H., Wacker, J. & Poreda, R. 3He in diamonds: The cosmogenic component. *Geochimica et Cosmochimica Acta* **53**, 569-574 (1989).

14 McConville, P., Reynolds, J., Epstein, S. & Roedder, E. Implanted 3He, 4He, and Xe in further studies of diamonds from Western Australia. *Geochimica et cosmochimica acta* **55**, 1977-1989 (1991).

15 Basu, S., Jones, A. P., Verchovsky, A. B., Kelley, S. P. & Stuart, F. M. An overview of noble gas (He, Ne, Ar, Xe) contents and isotope signals in terrestrial diamond. *Earth-Science Reviews* **126**, 235-249, doi:https://doi.org/10.1016/j.earscirev.2013.08.010 (2013).

16 Kramers, J. D. Lead, uranium, strontium, potassium and rubidium in inclusion-bearing diamonds and mantle-derived xenoliths from Southern Africa. *Earth and Planetary Science Letters* **42**, 58-70, doi:https://doi.org/10.1016/0012-821X(79)90190-0 (1979).

17 Lal, D. An important source of4He (and3He) in diamonds. *Earth and Planetary Science Letters* **96**, 1-7, doi:https://doi.org/10.1016/0012-821X(89)90118-0 (1989).

18 Luther, L. C. & Moore, W. J. Diffusion of Helium in Silicon, Germanium, and Diamond. *The Journal of Chemical Physics* **41**, 1018-1026, doi:10.1063/1.1726000 (1964).

19 Wiens, R. C., Lal, D., Rison, W. & Wacker, J. F. Helium isotope diffusion in natural diamonds. *Geochimica et Cosmochimica Acta* **58**, 1747-1757, doi:https://doi.org/10.1016/0016-7037(94)90534-7 (1994).

20 Orwa, J. O., Jamieson, D. N., Nugent, K. W., Prawer, S. & Kalish, R. Effects of damage on diffusion of implanted helium in diamond measured by nuclear elastic scattering. *Nuclear Instruments and Methods in Physics Research Section B: Beam Interactions with Materials and Atoms* **124**, 515-518, doi:https://doi.org/10.1016/S0168-583X(97)00086-4 (1997).

21 Zashu, S. & Hiyagon, H. Degassing mechanisms of noble gases from carbonado diamonds. *Geochimica et Cosmochimica Acta* **59**, 1321-1328, doi:https://doi.org/10.1016/0016-7037(95)00046-3 (1995).

22 Cherniak, D. J., Watson, E. B., Meunier, V. & Kharche, N. Diffusion of helium, hydrogen and deuterium in diamond: Experiment, theory and geochemical applications. *Geochimica et Cosmochimica Acta* **232**, 206-224, doi:https://doi.org/10.1016/j.gca.2018.04.029 (2018).

23 Shelkov, D. A., Verchovsky, A. B., Milledge, H. J. & Pillinger, C. T. The radial distribution of implanted and trapped 4He in single diamond crystals and implications for the origin of carbonado. *Chemical Geology* **149**, 109-116, doi:https://doi.org/10.1016/S0009-2541(98)00040-0 (1998).

24 Burgess, R., Johnson, L., Mattey, D., Harris, J., Turner, G. He, Ar and C isotopes in coated and polycrystalline diamonds. *Chemical Geology* **146**, 205-217 (1998).

25 Timmerman, S., Yeow, H., Honda, M., Howell, D., Jaques, A.L., Krebs, M.Y., Woodland, S., Pearson, D.G., Ávila, J.N., Ireland, T.R. U-Th/He systematics of fluid-rich ‘fibrous’ diamonds – Evidence for pre- and syn-kimberlite eruption ages. *Chemical Geology* **515**, 22-36 (2019).

26 Broadley, M. W. et al. Plume-lithosphere interaction, and the formation of fibrous diamonds. *Geochemical Perspectives Letters* **8**, 26-30, doi:http://dx.doi.org/10.7185/geochemlet.1825 (2018).

27 Timmerman, S., Honda, M., Phillips, D., Jaques, A. L. & Harris, J. W. Noble gas geochemistry of fluid inclusions in South African diamonds: implications for the origin of diamond-forming fluids. *Mineralogy and Petrology* **112**, 181-195, doi:10.1007/s00710-018-0603-x (2018).

28 Rege, S. et al. Trace-element patterns of fibrous and monocrystalline diamonds: Insights into mantle fluids. *Lithos* **118**, 313-337, doi:https://doi.org/10.1016/j.lithos.2010.05.007 (2010).

29 Schrauder, M., Koeberl, C. & Navon, O. Trace element analyses of fluid-bearing diamonds from Jwaneng, Botswana. *Geochimica Et Cosmochimica Acta* **60**, 4711-4724, doi:10.1016/s0016-7037(96)00274-8 (1996).
