# Supplementary material for: Helium in diamonds unravels over a billion years of craton metasomatism
Source: Nat Commun. 2021 May 11;12:2667. doi: 10.1038/s41467-021-22860-3 (PMC8113505; doi:10.1038/s41467-021-22860-3)
Supplement: Supplementary file 3 — Description of Additional Supplementary Files [file 41467_2021_22860_MOESM3_ESM.pdf]

Description of Additional Supplementary Files

File Name: Supplementary Data 1
Description: Helium content and isotopic composition, nitrogen aggregation and HDF compositional type in De Beers Pool and Finsch diamonds.

File Name: Supplementary Data 2
Description: Published He content and 3He/4He for CLM-derived xenoliths measured by crushing (as opposed to heating and fusion)
